# Estimating wildlife activity curves: comparison of methods and sample size

**DOI:** 10.1038/s41598-018-22638-6

**Published:** 2018-03-08

**Authors:** Marcus A. Lashley, Michael V. Cove, M. Colter Chitwood, Gabriel Penido, Beth Gardner, Chris S. DePerno, Chris E. Moorman

**Affiliations:** 1 Wildlife, Fisheries, and Aquaculture, Mississippi State University, Box 9690, Mississippi State, MS 39762 USA; 2 Department of Applied Ecology, North Carolina State University, Raleigh, North Carolina 27695 USA; 3 Wildlife Biology Program, University of Montana, Missoula, MT 59812 USA; 4 Programa de Pós-Graduação em Ecologia, Departamento de Zoologia, Universidade de Brasília, Darcy Ribeiro, 70910-900 Brazil; 5 Environmental and Forest Sciences, University of Washington, Seattle, WA 98195 USA; 6 Fisheries, Wildlife, and Conservation Biology Program, North Carolina State University, Raleigh, NC 27606 USA

## Abstract

Camera traps and radiotags commonly are used to estimate animal activity curves. However, little empirical evidence has been provided to validate whether they produce similar results. We compared activity curves from two common camera trapping techniques to those from radiotags with four species that varied substantially in size (~1 kg–~50 kg), diet (herbivore, omnivore, carnivore), and mode of activity (diurnal and crepuscular). Also, we sub-sampled photographs of each species with each camera trapping technique to determine the minimum sample size needed to maintain accuracy and precision of estimates. Camera trapping estimated greater activity during feeding times than radiotags in all but the carnivore, likely reflective of the close proximity of foods readily consumed by all species except the carnivore (i.e., corn bait or acorns). However, additional analyses still indicated both camera trapping methods produced relatively high overlap and correlation to radiotags. Regardless of species or camera trapping method, mean overlap increased and overlap error decreased rapidly as sample sizes increased until an asymptote near 100 detections which we therefore recommend as a minimum sample size. Researchers should acknowledge that camera traps and radiotags may estimate the same mode of activity but differ in their estimation of magnitude in activity peaks.

## Introduction

Understanding diel activity is fundamental to understanding the ecology and management of wildlife species. For example, activity curves (i.e., activity patterns) inform the movement ecology that regulates physiology of individuals and growth of a population^1^ and the decision rules animals follow given tradeoffs associated with forage acquisition, thermoregulation, and predator avoidance^2^. More recently, activity curve relationships between species have been used to address hypotheses concerning interspecific and intraspecific competition and predator-prey interactions^3–7^. Because of their importance, a variety of methods have been proposed to estimate activity curves^8^.

Historically, visual observations in the wild^9^ or in laboratory settings^10^ were used to determine the activities of an animal species. Visual observations in the wild have obvious flaws related to concealment of the observer and detection of the subject, and observations in labs can result in altered behaviors that preclude their widespread usefulness in determining activities of wild conspecifics. However, radiotags have allowed the collection of precise and frequent animal relocations, thus allowing more accurate monitoring of animal activity in the wild^11^. Moreover, fitting animals with radiotags allows frequent relocation of individuals, which translates to the accumulation of relatively large datasets that can be retrieved remotely and in near real time^11^. But, physically tagging animals is an invasive, expensive, labor intensive, and in some cases unfeasible sampling technique (e.g., elusive or rare animals, endangered species). Moreover, Rowcliffe *et al*.^12^ reported that multiple relocations per minute would be required to accurately measure movement distances, which is an important measure to calculate activity patterns from radiotag data. Because radiotags are commonly used to test hypotheses not related to activity patterns, and often those questions require long term data sets that do not allow such frequent relocation, very few studies have relocations more frequent than even every few hours^12^. Nonetheless, radiotags increasingly are being used to estimate activity curves to evaluate a variety of hypotheses on a wide variety of species (e.g.,^13–17^).

Camera traps have the potential advantage to inexpensively and noninvasively sample wildlife populations. The equipment is relatively cheap, and the technique does not rely on animal capture, which results in lower cost and labor effort^18^. Also, camera trapping may provide a means of gathering large datasets on elusive, rare, and protected species^19,20^, which can be used for a wide range of purposes^21,22^. For example, camera traps have been used as a research tool to discover new species^23^, conduct simple vertebrate inventories^24^, estimate density^25^, measure population dynamics^26^, examine habitat associations of entire animal communities^27^, evaluate hypotheses about interspecific interactions^28^, assess animal behavior^7,29^, and estimate activity curves^8,18^. Thus, the versatility of camera traps has led to an explosion in their use^21^ and advancement in analytical techniques^8,30^.

One common use of camera traps and radiotags is to monitor wildlife activity curves, so there is a need to test whether these methods produce similar results. The fundamental difference in the way the data are collected between the methods indicates that some differences in the resulting activity curves may exist. That is, camera traps are detecting all individuals that occur at a single area, whereas radiotags continually sample a subset of individuals but across areas. In some cases, camera traps may only detect animals when moving outside of cover^8^, which could result in biases in activity curves if animals are active while in areas not being sampled, a problem not present with radiotags. Also, the accuracy of camera traps in determining the activity curves may be affected by imperfect detection, and it is unknown if the size, feeding ecology, or activity mode of the targeted species affect the accuracy of activity curve estimation. Ridout & Linkie^31^ tested differing modes of activity when performing overlap analyses, but empirical data are needed to test if differing modes of activity, differential detectability associated with body size^20^, or feeding ecology affects activity curve estimates. Moreover, the camera trapping design may affect the reliability of activity curve estimates. Rowcliffe *et al*.^8^ suggested using randomly placed camera traps when estimating activity curves, but the reality is that the two most common trapping methods (i.e., baited sites - hereafter active method and placement on trails - hereafter passive method) are designed to maximize the detection probability of elusive species^21^ and estimating activity curves is a secondary objective to maximizing detections.

The number of animal detections may influence the reliability of subsequent activity curve estimates^8^. Ridout & Linkie^31^ conducted analyses on sample sizes as small as 25 detections to test overlap analyses and Rowcliffe *et al*.^8^ reported 100 detections were necessary for accuracy, but subsequent studies have created activity curves from fewer detections (e.g., 4 and 8 detections^32^, 14 detections^6^, 18 detections^33^). It is not clear how accurate the activity curve estimates are at such small sample sizes, whether necessary sample sizes are influenced by passive or active sampling methodologies, or how they compare to radiotag estimates.

**Figure 1:**
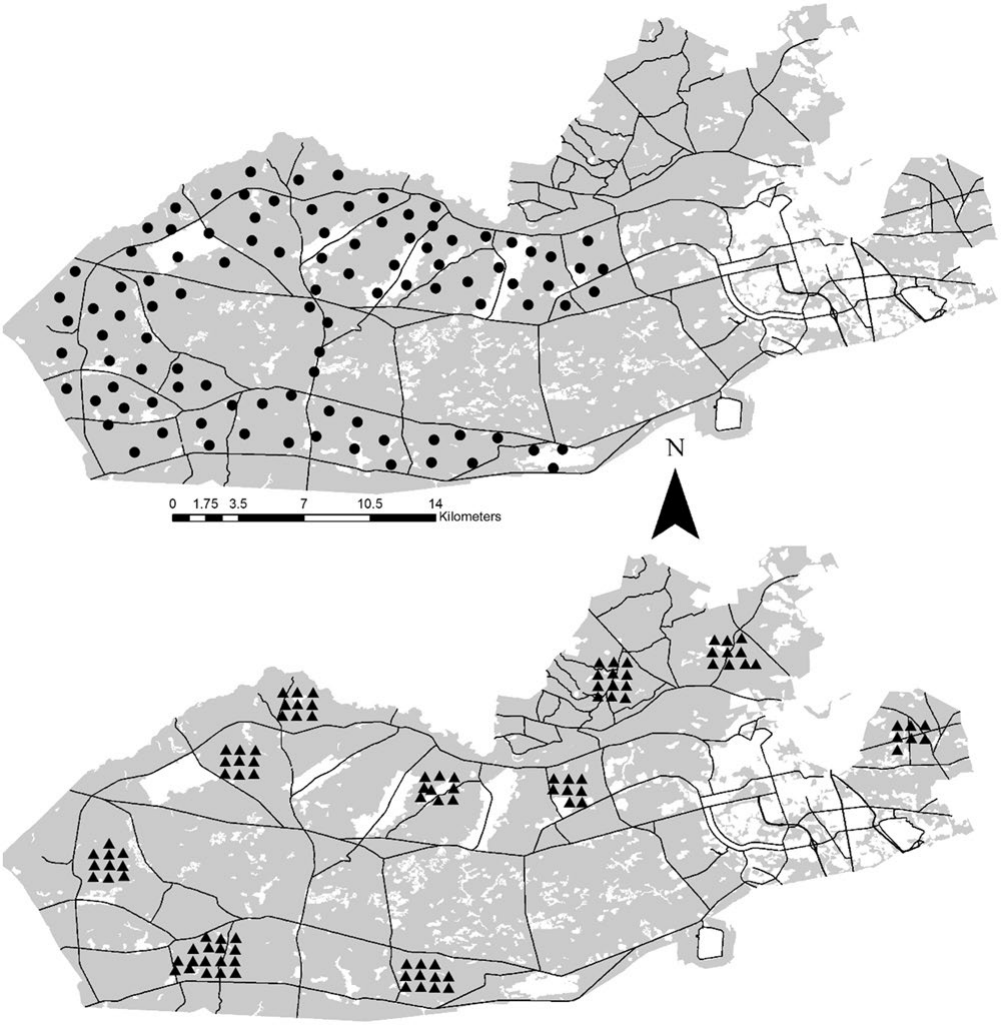
Locations of active (triangle) camera traps in August 2011–2012 and passive (circle) camera traps in January-March 2012 at Fort Bragg Military Installation, North Carolina, USA. The map was created using ArcMap 10.3.1 (https://www.esri.com/).

We simultaneously deployed camera traps (Fig. 1) in a baited (active camera trapping) and trail-targeted (passive camera trapping) design in an area with 4 species of radiotagged vertebrates of varying size, mode of activity, and feeding ecology, which allowed us to compare common camera trapping methods to radiotags in terms of the activity curves they produced. Further, we sub-sampled detections at camera traps to determine at what sample size the accuracy and precision of estimates became unreliable and whether species ecology or camera design influenced reliability.

## Results

### Summary Statistics

During 2,800 trap nights, active camera traps recorded 306 detections of coyotes (0.11 detections/trap night), 104 detections of fox squirrels (0.04 detections/trap night), 237 detections of hen wild turkeys (0.08 detections/trap night), and 39,586 detections of male and female deer (14.14 detections/trap night). During 7,700 trap nights, passive camera traps recorded 983 detections of coyotes (0.13 detections/trap night), 83 detections of fox squirrels (0.01 detections/trap night), 99 detections of hen wild turkeys (0.01 detections/trap night), and 163 detections of male and female deer (0.02 detections/trap night). Thus, active camera trapping tended to be more efficient than passive camera trapping for photo-capturing fox squirrels, wild turkeys, and deer, and tended to be similarly efficient for coyotes.

### Comparison of Radiotags and Camera Traps

**Figure 2:**
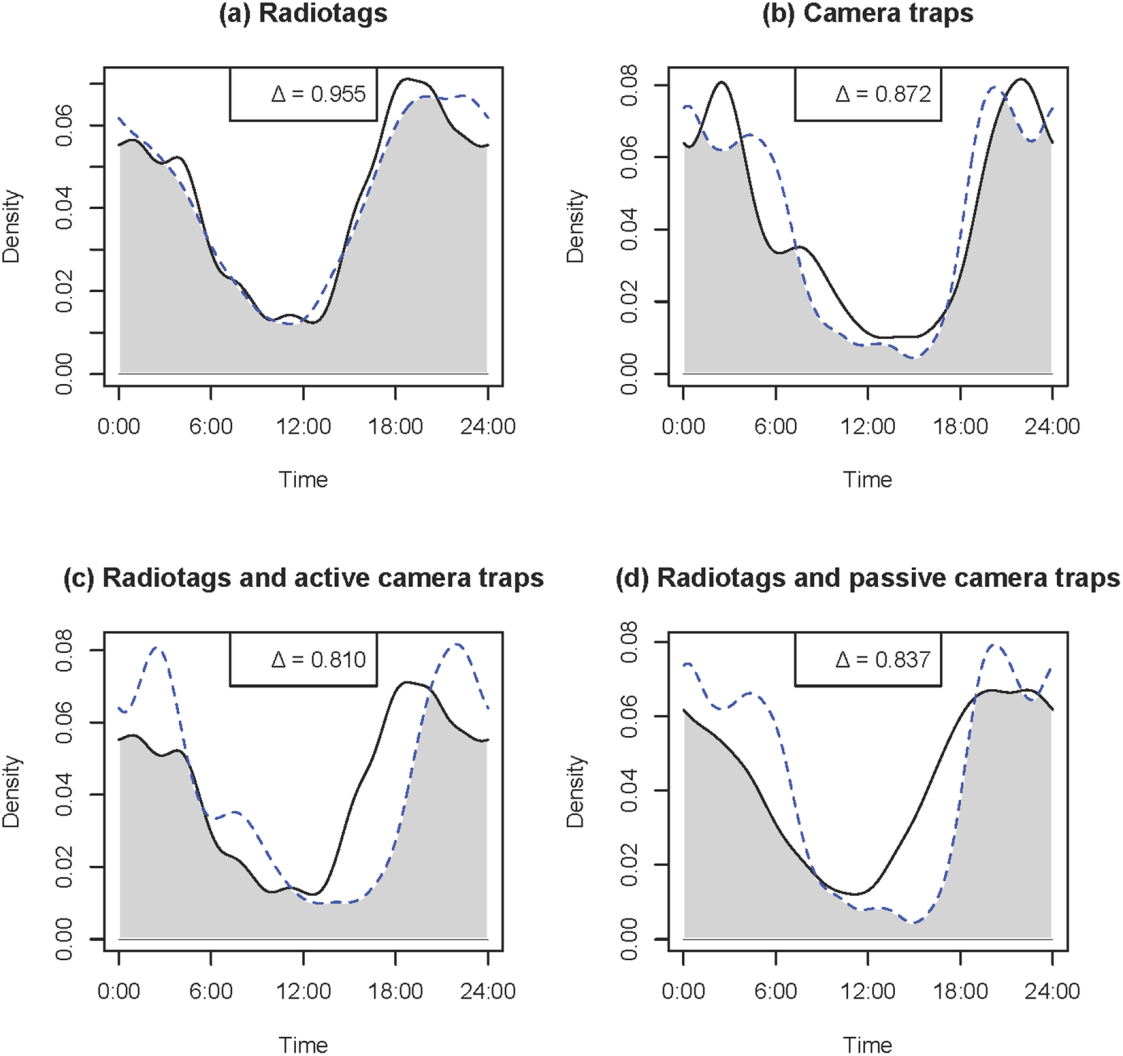
Overlap plots of the activity curves of coyotes *Canis latrans* estimated from (**a**) radiotags during August (solid line) and January-March (dotted line) (Δ = 0.955), (**b**) active (solid line) and passive (dotted line) camera traps (Δ = 0.872), (**c**) radiotag (solid line) and active camera traps (dotted line) (Δ = 0.810), and (**d**) radiotags (solid line) and passive camera traps (dotted line) (Δ = 0.837) at Fort Bragg, NC, 2011–2012.

**Figure 3:**
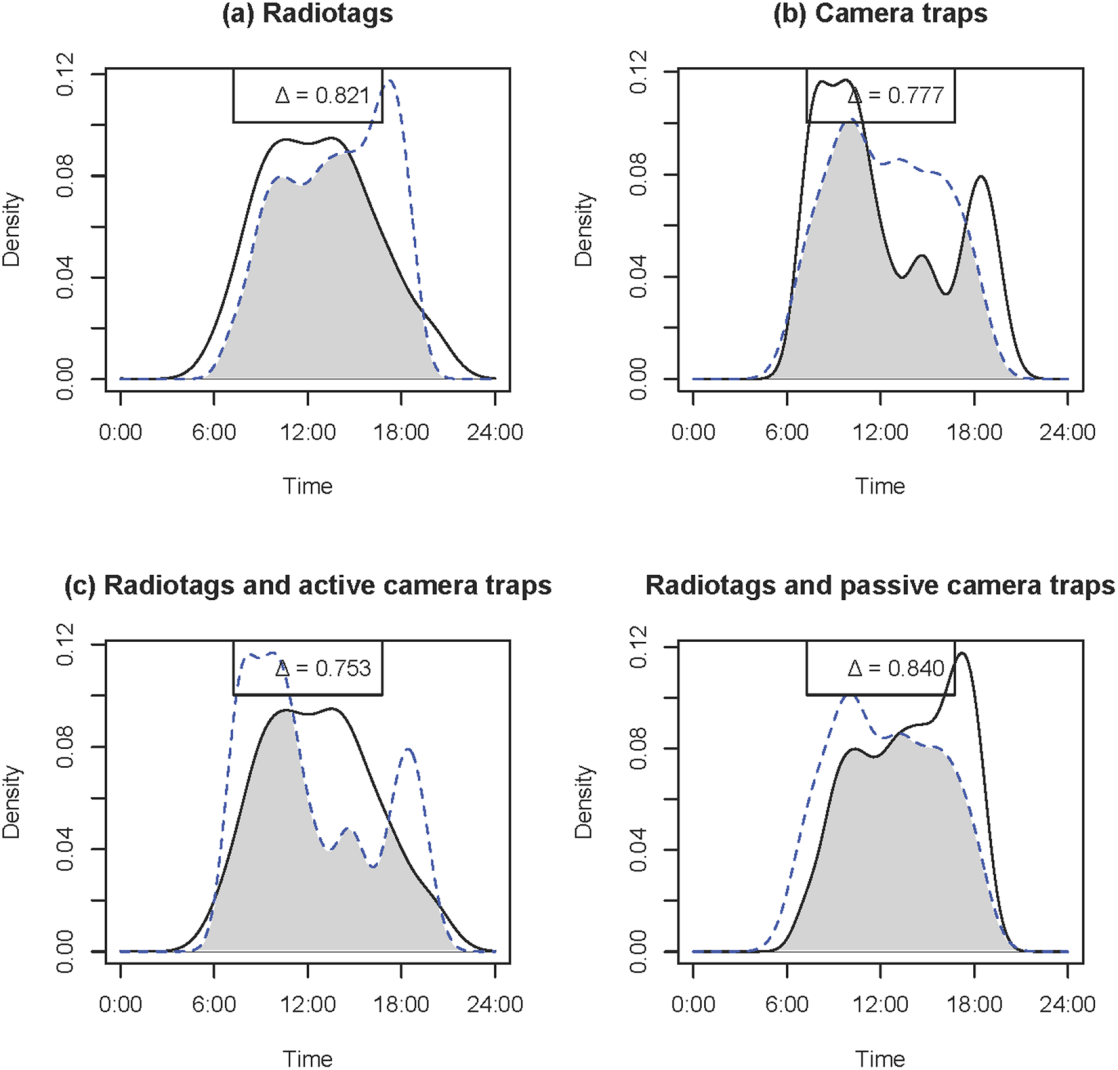
Overlap plots of the activity curves of southeastern fox squirrel *Sciurus niger niger* estimated from (**a**) radiotags during August (solid line) and January-March (dotted line) (Δ = 0.821), (**b**) active (solid line) and passive (dotted line) camera traps (Δ = 0.777), (**c**) radiotag (solid line) and active camera traps (dotted line) (Δ = 0.753), and (**d**) radiotags (solid line) and passive camera traps (dotted line) (Δ = 0.840) at Fort Bragg, NC, 2011–2012.

**Figure 4:**
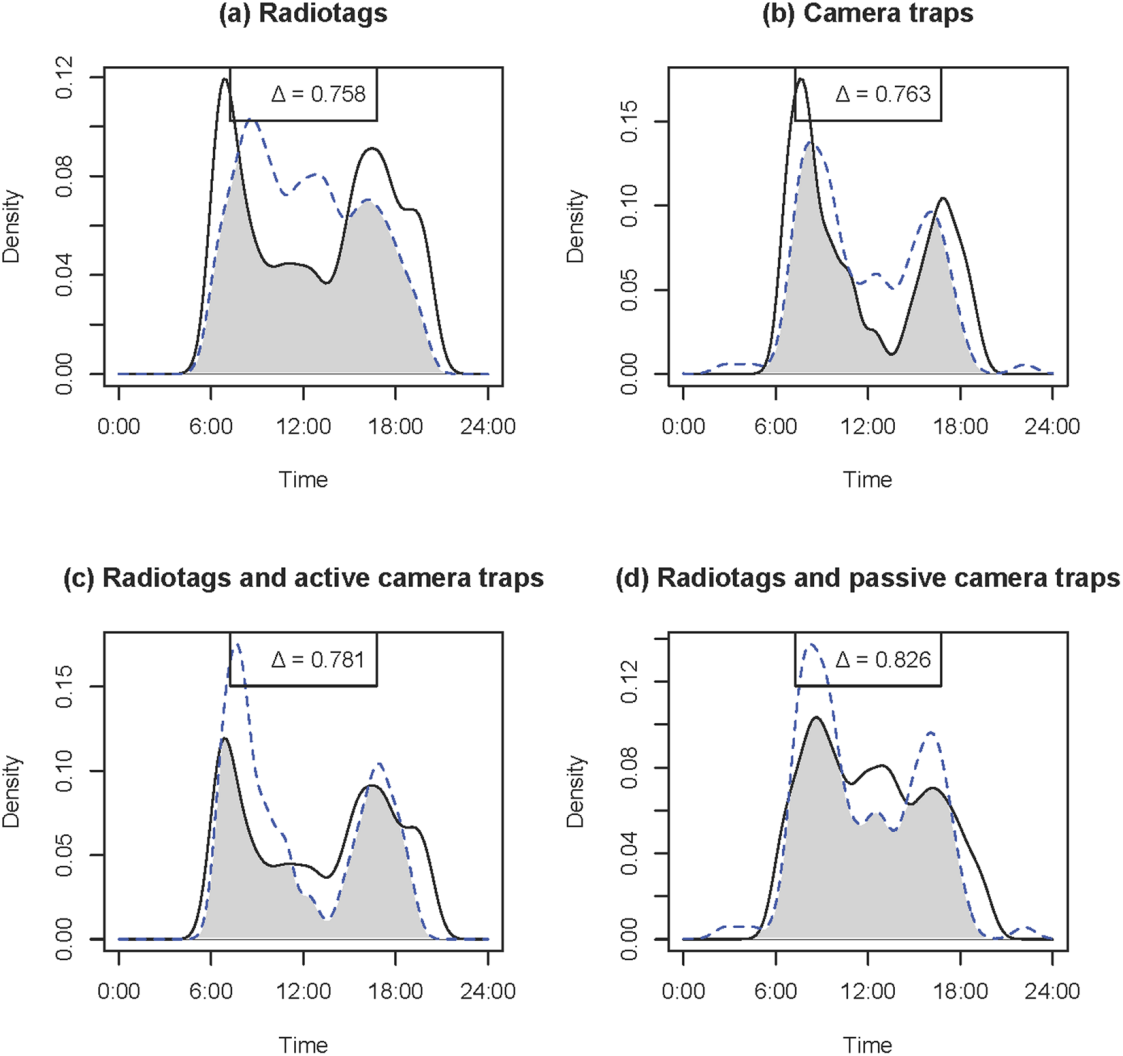
Overlap plots of the activity curves of wild turkey *Meleagris gallopavo* estimated from (**a**) radiotags during August (solid line) and January-March (dotted line) (Δ = 0.758), (**b**) active (solid line) and passive (dotted line) camera traps (Δ = 0.763), (**c**) radiotag (solid line) and active camera traps (dotted line) (Δ = 0.781), and (**d**) radiotags (solid line) and passive camera traps (dotted line) (Δ = 0.826) at Fort Bragg, NC, 2011–2012.

**Figure 5:**
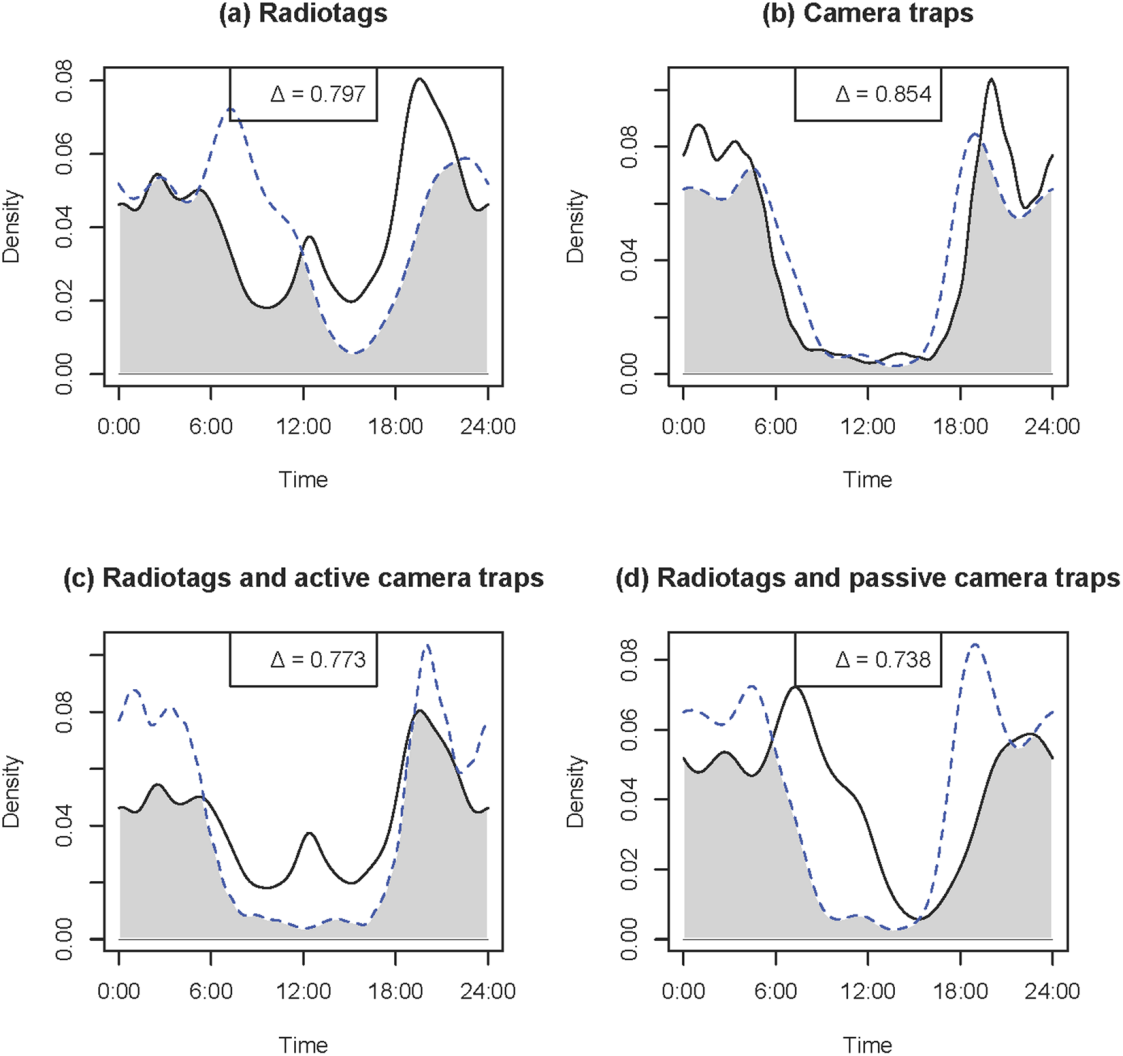
Overlap plots of the activity curves of white-tailed deer *Odocoileus virginianus* estimated from (**a**) radiotags in during August (solid line) and January-March (dotted line) (Δ = 0.797), (**b**) active (solid line) and passive (dotted line) camera traps (Δ = 0.854), (**c**) radiotag (solid line) and active camera traps (dotted line) (Δ = 0.773), and (**d**) radiotags (solid line) and passive camera traps (dotted line) (Δ = 0.738) at Fort Bragg, NC, 2011–2012.

Seasonal activity curve overlap based on the radiotags ranged from Δ = 0.758 (wild turkeys) to Δ = 0.955 (coyotes) (Table 1; Panel a, Figs 2–5). Similarly, seasonal activity curve overlap between active and passive camera traps ranged from Δ = 0.763 (wild turkeys) to Δ = 0.872 (coyotes) (Table 1; Panel b, Figs 2–5). Thus, both methods were able to estimate a similar shift in activity, which may be linked to shifts in resource availability, environmental conditions, day length, competition, predation or other interactions of interest in ecology. When comparing active camera trap-derived activity curves to radiotag-derived activity curves, estimated coefficients of overlap were relatively high (i.e., Δ = 0.753–0.810) and coefficients were correlated (i.e., R = 0.66–0.85), but the Watson’s U2 test indicated active camera trap-derived estimates were different from the radiotag-derived estimates for all species (Table 1; Panel c, Figs 2–5). When comparing passive camera trap-derived activity curves to radiotag-derived activity curves, estimated coefficients of overlap also were high (i.e., Δ = 0.738–0.840) and coefficients were correlated (i.e., R = 0.41–0.91), but the Watson’s U2 test indicated passive camera trap-derived estimates were significantly different from the radiotag-derived estimates for deer and coyotes (Table 1; Panel d, Figs 2–5). When compared to radiotag-derived activity curves, passive camera traps provide a slightly more similar estimate than active camera traps (active mean Δ = 0.78, passive mean Δ = 0.81), but active camera trapping was slightly more precise than passive camera trapping (active range Δ = 0.75–0.81, passive range Δ = 0.74–0.84).

**Table 1:** Coefficient of overlap (∆ with 95% bootstrapped confidence intervals), coefficient of determination (R), and Watson’s U2 statistic (U2) comparing active and passive camera trapping techniques to estimate animal activity curves to estimates derived from radiotag data on 4 species, Fort Bragg Military Installation, North Carolina, USA, 2011–2012.

Species	Test^a^	Active X Radiotag^b^	Passive X Radiotag^b^	Radiotag Comparison^c^	Camera Comparison^d^
Coyote^a^	∆	0.81 (0.75–0.83)	0.84 (0.81–0.86)	0.96 (0.94–0.97)	0.87 (0.82–0.89)
	R	0.66 (p < 0.001)	0.79 (p < 0.001)	0.97 (p < 0.001)	0.90 (p < 0.001)
	U2	1.13 (p < 0.001)	2.11 (p < 0.01)	0.38 (p < 0.001)	0.14 (p > 0.10)
Fox squirrel	∆	0.75 (0.65–0.84)	0.84 (0.77–0.96)	0.82 (0.75–0.91)	0.78 (0.67–0.88)
	R	0.74 (p < 0.001)	0.87 (p < 0.001)	0.85 (p < 0.001)	0.79 (p < 0.001)
	U2	0.43 (p < 0.001)	0.12 (p > 0.10)	0.24 (p < 0.05)	0.27 (p < 0.01)
Wild turkey	∆	0.78 (0.74–0.85)	0.83 (0.73–0.89)	0.76 (0.70–0.77)	0.76 (0.69–0.86)
	R	0.84 (p < 0.001)	0.91 (p < 0.001)	0.73 (p < 0.001)	0.83 (p < 0.001)
	U2	0.73 (p < 0.001)	0.11 (p > 0.10)	3.27 (p < 0.001)	0.43 (p < 0.001)
White-tailed deer	∆	0.77 (0.75–0.80)	0.74 (0.66–0.77)	0.80 (0.76–0.82)	0.85 (0.81–0.91)
	R	0.85 (p < 0.001)	0.41 (p < 0.001)	0.33 (p < 0.001)	0.88 (p < 0.001)
	U2	10.6 (p < 0.001)	0.67 (p < 0.01)	3.09 (p < 0.001)	0.33 (p < 0.01)

^a^Significant p-values indicate significant correlation for the coefficient of determination and indicate the methods are significantly different for the Watson’s U2 statistic. Alpha = 0.05.

^b^Comparison of activity curve estimates derived from the camera trapping method and respective radiotags.

^c^Comparison of the radiotag-derived activity curves estimates during each sampling period for the respective species.

^d^Comparison of the camera trap-derived activity curve estimates from each trapping design for the respective species.

### Comparison of Sample Sizes

**Figure 6:**
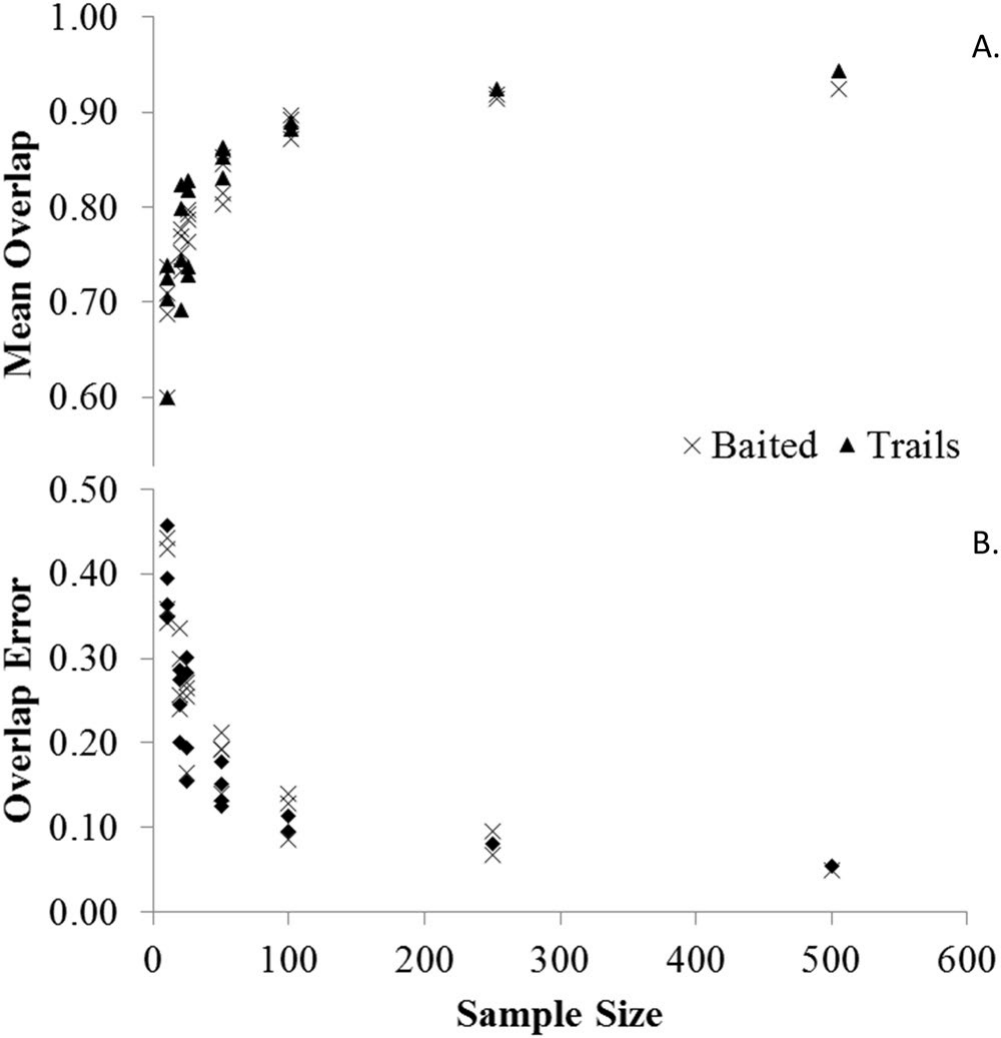
Estimated coefficients of overlap (**A**) and associated 1000-iteration bootstrapped estimates of error (**B**) using active and passive camera trap detection sample sizes of coyotes *Canis latrans*, southeastern fox squirrel *Sciurus niger niger*, wild turkey *Meleagris gallopavo*, and white-tailed deer *Odocoileus virginianus* at Fort Bragg, NC, 2011–2012.

Among all species, sub-sampling of active and passive camera trap detections revealed an overall trend of decreasing Δ and increasing error as the sub-sample size decreased (Fig. 6). Sub-samples remained significantly correlated (R ≥ 0.56) and were not significantly different from the overall dataset for all species for both camera trapping methods with as few as 10 detections, with the exception of coyotes at the 250 sub-sample level of passive camera trap-derived estimates (Table 2). However, an asymptote emerged for Δ and associated 95% confidence interval widths at ~100 detections, where increasing sample size yielded little overall improvement in accuracy or precision of activity curve estimates (Fig. 6).

**Table 2:** Coefficient of overlap (∆), coefficient of determination (R), and Watson’s U2 statistic (U2) comparing activity curve estimates (derived from 7 sub-sample sizes of camera detections) to the estimates derived from the respective complete dataset of active and passive camera trapping detections of 4 species, Fort Bragg Military Installation, North Carolina, USA, 2011–2012.

Species	Method	Test	500	250	100	50	25	20	10
Coyote	Active	∆		0.97	0.89	0.87	0.83	0.8	0.67
		R		0.99*	0.95*	0.96*	0.80*	0.78*	0.63*
		U2		0.007	0.07	0.05	0.09	0.10	0.06
	Passive	∆	0.97	0.91	0.91	0.87	0.86	0.84	0.71
		R	0.99*	0.94*	0.95*	0.93*	0.91*	0.87*	0.77*
		U2	0.02	0.21*	0.09	0.04	0.02	0.05	0.07
Fox squirrel	Active	∆			0.98	0.88	0.93	0.73	0.79
		R			0.99*	0.94*	0.97*	0.8*	0.85*
		U2			0.003	0.06	0.03	0.12	0.05
	Passive	∆				0.92	0.89	0.9	0.71
		R				0.97*	0.94*	0.95*	0.76*
		U2				0.03	0.03	0.02	0.06
Wild turkey	Active	∆			0.94	0.88	0.8	0.78	0.78
		R			0.98*	0.96*	0.91*	0.84*	0.83*
		U2			0.02	0.08	0.05	0.06	0.06
	Passive	∆				0.94	0.87	0.83	0.84
		R				0.99*	0.92*	0.90*	0.91*
		U2				0.01	0.03	0.05	0.02
White-tailed deer	Active	∆	0.94	0.98	0.9	0.91	0.88	0.82	0.79
		R	0.98*	0.97*	0.94*	0.96*	0.91*	0.9 *	0.83 *
		U2	0.11	0.05	0.03	0.04	0.02	0.08	0.05
	Passive	∆			0.94	0.85	0.9	0.74	0.62
		R			0.98*	0.89*	0.94*	0.69*	0.56*
		U2			0.02	0.07	0.03	0.13	0.13

*Denotes significance at alpha = 0.05. Blank spaces were not tested because the sample size was not available.

## Discussion

The majority of dissimilarity in camera trap- and radiotag-derived activity curves resulted from the magnitude of peaks in feeding activity (i.e., crepuscular times). As a result, passive camera traps tended to be more similar to radiotags than active camera traps, probably because animals seek out bait more intensely while actively feeding and both radiotags and passive camera traps are more likely to detect other activities. This effect may have been more pronounced if acorns (*Quercus* spp.), an important food source for each species except coyotes^34^, were not disproportionately concentrated near trails at the study area during the passive camera trap sampling period^35^. As additional evidence that the dissimilarity of feeding times was a result of proximate food sources, coyotes did not readily consume corn or acorns at Fort Bragg^36^, and as a result, active and passive camera trap-derived activity curves did not consistently estimate a greater magnitude during peak feeding activity as compared to radiotags. However, we also acknowledge that radiotags could have underestimated peaks relative to camera traps if relative tortuosity (i.e., measure of deviation from linear) of movements increased near feeding times^12^. In that case, an alternative explanation is that the feeding behavior of the predator did not yield the same changes in tortuosity during feeding times as the other species studied.

Active camera traps were more precise than passive camera traps likely because of the greater trapping efficiencies associated with bait intentionally placed in the camera trap sampling area. This effect was most pronounced in deer with the 700-fold greater detection rate with active versus passive camera traps. The substantially greater sample size increased the accuracy of camera trap-derived activity curve estimates by 0.04, which is a substantial increase given that there were more than 100 detections (i.e., the detection threshold of diminishing return in accuracy and precision with sample size) on passive camera traps. The increased sample sizes likely had the same effect on the precision of estimates, based on the sub-sampling exercise we performed on camera trap datasets. Therefore, the use of lures might be useful in some cases because of the increase in detections per trap night, particularly when inferring the activity curves of rare or elusive study species or when available sampling periods and manpower are limited, assuming lures do not otherwise introduce bias to activity.

Using the appropriate camera trapping technique to target species based on their ecology is important to maximize the accuracy of activity curve estimates. For example, the passive camera traps (i.e., targeted coyotes) tended to estimate coyote activity curves more similar to radiotags, whereas active camera traps (i.e., targeted deer) tended to provide more similar activity curves to radiotags in deer. Each method targeted the ecology of the respective species and as a result led to more similar activity curve estimates between cameras and radiotags. For example, white-tailed deer are herbivores that seek out plant parts high in energy, particularly during the late-summer stress period when resources are scarce^37^, which we exploited by baiting sites with corn in the active camera trapping design. Likewise, the corn attractant proved effective to survey fox squirrels and wild turkeys that also readily consumed the bait. Contrastingly, the omnivorous coyotes were not attracted to bait, which we anticipated because of their food habits^36^; thus, we exploited the coursing hunting behavior prevalent in canids by placing camera traps on trails in the passive camera trap design. Because coyotes are coursing predators, they typically travel long distances daily in search of food and likely use trails to conserve energy in the process^38^.

Interestingly, sub-samples from the camera trap datasets revealed a clear asymptote where increasing sample size did not increase accuracy or precision despite the life history traits of the species that may affect their detection probability (e.g., body size, movement rate^8,39^). Although Rowcliffe *et al*.^8^ reported a diminishing return at the same sample size, we add that the style of camera trapping also did not influence the detection threshold, indicating 100 detections may be a widely applicable guideline to future studies that estimate wildlife activity curves with camera trap data collected in a variety of manners. Moreover, care should be taken when interpreting activity curves generated from smaller sample size data sets because of the rapidly diminishing accuracy and precision of estimates, despite their correlation and Watson’s U2 suggesting similarities. Therefore, similar to Rowcliffe *et al*.^8^, we recommend researchers collect at least 100 detections of a targeted species before estimating activity curves from camera trap data. The effort needed to obtain 100 detections, however, may vary widely depending on the ecology, mobility, and detectability of the species^39^. Moreover, as we demonstrated herein, wildlife species commonly shift activity patterns seasonally, so active camera trapping may be a requirement in some cases to acquire a robust sample size within a timeframe relevant to the life history of the species studied. Thus, the ecology of the species is a key consideration when designing camera trap studies. We caution users of these methodologies to be clear about the strengths and limitations of the methods chosen, particularly when using lures or trail-targeting, and strive to collect adequate sample size to make robust inferences.

## Methods

We conducted our study in 2011 and 2012 at Fort Bragg Military Installation (~65,000 ha; hereafter Fort Bragg), located in the Sandhills physiographic region in southeastern North Carolina, USA (358170 N, 828470 W). Roughly 65% of Fort Bragg was forested and ~35% was unforested openings. Fort Bragg maintained a well-developed firebreak system, which facilitated large-scale implementation of growing-season prescribed fire on a 3-yr fire-return interval^35^. Firebreaks (hereafter trails) were oriented east-to-west and spaced ~200 m apart, with intermittent north-to-south trails^35^.

### Coyote

During February – May 2011, we captured 30 coyotes *Canis latrans* with MB-550 foothold traps^40^. We deployed Wildcell SG GPS radiotags (Lotek Wireless Inc., Ontario, Canada) that were programmed to record a GPS location every 3 hours^40^. Mean weight of radiotagged coyotes was 12.9 kg (M. Swingen, unpublished data). Studies of coyote activity curves have indicated they are largely crepuscular and nocturnal (e.g.,^41,42^), despite evidence that their visual system is most likely adapted to diurnal and crepuscular activity^43^. All capture and handling protocols were approved by the North Carolina State University (NCSU) Institutional Animal Care and Use Committee (IACUC; #11–005-O) and all experiments were performed in accordance with relevant guidelines and regulations.

### Southeastern fox squirrel

During January – May 2011, we trapped southeastern fox squirrels *Sciurus niger niger* L. using wooden box traps and wire-cage traps (Tomahawk Live Trap Company, Tomahawk, Wisconsin, USA) baited with dried whole kernel corn^44^. We radiotagged (Model SI-2C, Holohil Sys. Ltd., Ontario, Canada) 33 adult fox squirrels weighing 0.75–1.25 kg with VHF radiotags. Because fox squirrels are known to be exclusively diurnal^45^, we relocated them at random times between sunrise and sunset once per day and at least 3 times per week. The day was broken into 3–4hr categories (morning, midday, and afternoon) and relocations were stratified across these time periods for each squirrel to ensure equal sampling effort throughout the day. We assessed the activity status (based on the emitted VHF cadence) of an individual before approaching for a relocation^44,46^. All capture and handling protocols were approved by the NCSU IACUC (#10-153-O) and all experiments were performed in accordance with relevant guidelines and regulations.

### Wild turkey

During February – April 2011, we captured female wild turkeys *Meleagris gallopavo* L. by rocket-netting^47,48^. We fitted 8 turkeys with 85-g GPS radiotags (Model G1H271 Sirtrack LTD, Havelock North, New Zealand) that were programmed to obtain 4 relocations daily during daylight hours (with one near dawn and dusk) because turkeys are diurnal^49^. Hunter harvest data on site indicated female turkeys weighed ~6 kg on average. All capture and handling protocols were approved by the North Carolina Wildlife Resources Commission and the NCSU IACUC (#10-149-A) and all experiments were performed in accordance with relevant guidelines and regulations.

### White-tailed deer

During January-June 2011, we used tranquilizer guns to capture 30 female white-tailed deer *Odocoileus virginianus* Zimmerman ≥1.5-years-old and fit them with 200-g GPS radiotags (Wildcell, Lotek Wireless Inc., Newmarket, Ontario, Canada) set to relocate individuals every 2.5 hours. Hunter harvest data on site indicated adult male and female deer weighed between 35 and 65 kg on average^50^. Deer activity curves have been reported as strongly bimodal (i.e., crepuscular^51^). All capture and handling protocols were approved by the North Carolina Wildlife Resources Commission and the NCSU IACUC (#10-143-O) and all experiments were performed in accordance with relevant guidelines and regulations.

### Camera Trapping

We established active (i.e., baited) camera traps during August (i.e., summer) of 2011 and 2012^29^ and passive (i.e., trail monitoring) camera traps on trails during January-March (i.e., winter) of 2012 (Fig. 1). For the active camera trapping, we established 100 camera trap locations in a systematically random distribution across Fort Bragg. We baited the locations with corn, which is readily consumed by all of the captured species except coyotes. For the passive camera trapping, we systematically placed camera traps at 101 locations along trails across Fort Bragg spaced 1.5 km apart on average, leaving them unbaited and activated for about 5 weeks at each site. Both active and passive camera trap delays were set so that passing animals could be detected as frequently as every 3 minutes. We only used female turkeys from camera traps because we only radiotagged females. However, we used both sexes for deer because a recent study in this deer population demonstrated that males and females have similar overall activity patterns^7^.

### Modeling

We determined the activity curves for each species from radiotag-derived relocation data and detections in camera trap surveys. We only used radiotag information that was collected concurrent with camera trap surveys (i.e., during summer or winter of the same year). This allowed us to make specific comparisons between active and passive camera trap-derived activity curves and radiotag-derived activity curves for each species over the same time period. To determine each species’ activities from these methods, we used the nonparametric kernel density approach suggested by Ridout & Linkie^31^. In these models, the camera trap detections were considered random samples from a continuous distribution (i.e., time of day) over the course of a 24-hr period, which was then used to estimate a probability density function (PDF). For the radiotag information, we used the movement rate per hour (i.e., distance between consecutive points divided by the hours between) for GPS radiotags and the bimodal activity sensor from the VHF radiotags on fox squirrels. To be comparable to camera trap-derived activity curves, the movement rates needed to be converted to a continuous variable over a 24-hr period. Thus, we weighted radiotag-derived inputs for each hour by its associated movement rate for GPS radiotagged species. For example, for a 125 m/hr movement rate for 0100–0200, we randomly distributed 125 inputs (i.e., equivalent to camera trap detection) between 0100 and 0200. This allowed the data to be continuous but still representative of the observed activity curves based on movement rates. An assumption implicit to this approach is that animals were active the entire time between points, which may not always be true. However, we believe our approach is conservative because we know the minimum distance the animal travelled between successive relocations and redistributed data with an equal likelihood within the timeframe it occurred. Fox squirrel inputs were each time that the activity sensor cadence indicated the animal was active, which is similar to the way camera trap inputs were tallied. However, it should also be noted that the motion trigger on the fox squirrel radiotag monitored body movement which was different from camera traps that monitor time of presence at the location or radiotags that monitor distances moved in a time interval. Thus, active fox squirrels could be in a nest and not necessarily moving any distance in space and thus, results should be interpreted accordingly. Moreover, we pooled movement rates from radiotags across animals for each species because that is the approach taken with camera traps in unmarked populations. Once a PDF was established for each species with each sampling technique, we calculated the coefficient of overlap (Δ), a continuous variable between 0 and 1 (where 1 represents identical curves). This coefficient is defined as the area under the curve formed by taking the minimum of each density function of the two compared cycles at each time point^52^. Ridout & Linkie^31^ showed that in simulation studies, the estimator Δ_1_ performed better with smaller datasets while Δ_4_ was more adequate for large datasets. We conducted the analyses in the R package ‘overlap’ (R community^53^). We used 1000 bootstrapped samples from each distribution to estimate the 95% confidence interval of Δ for each comparison. The coefficient of overlap is descriptive, without a clear indication of a threshold value below which two activity curves are significantly different. Therefore, we applied the Watson’s U2 statistic^54^ implemented in the package ‘CircStats’^55^. This test computes the probability that two samples of circular data come from the same population, i.e., if they are homogeneous or not. Finally, we sub-sampled from the PDFs to estimate correlation coefficients of determination (e.g., Pearson’s R) in R (R community^53^). We made four intraspecific comparisons for each of the four species in the analysis. We determined the overlap (i.e., Δ) and similarity (i.e., Watson’s U2), and then correlated (i.e., R) the PDFs from: 1) the two seasons of radiotag-derived activity curves; 2) the two seasons of camera trap-derived activity curves; 3) the active camera trap-derived activity curves and radiotag-derived activity curves; and 4) the passive camera trap-derived activity curves and radiotag-derived activity curves.

We determined the necessary sample size of camera trap detections to accurately and precisely estimate activity curves for each species with active and passive camera trapping. For each species, we randomly sub-sampled the full active and passive camera trap datasets. We obtained sub-samples of 500, 250, 100, 50, 25, 20, and 10 detections, where available (i.e., sub-samples were dependent on the total number of detections). We then estimated the PDF for each sub-sample to estimate a coefficient of overlap with the full dataset. To account for sample size in the model, we chose to use the Δ_1_ overlap coefficient when sample sizes were less than 50 and Δ_4_ for samples greater than 50^53^. Within the ‘overlap’ package, we performed 1000 bootstraps to estimate a mean coefficient of overlap and 95% confidence intervals. We performed interval corrections on a logistic scale and back-transformed them to correct for confidence intervals if estimates fell outside of the possible range of 0–1^53^. We plotted Δ estimates and also the associated 95% confidence interval widths to determine the relationship between sample size and accuracy and precision compared to full data sets for each species. The datasets generated during and analysed during the current study are available from the corresponding author on reasonable request.
